# Tuned S-Scheme Cu_2_S_TiO_2__WO_3_ Heterostructure Photocatalyst toward S-Metolachlor (S-MCh) Herbicide Removal

**DOI:** 10.3390/ma14092231

**Published:** 2021-04-26

**Authors:** Alexandru Enesca, Luminita Isac

**Affiliations:** 1 Product Design, Mechatronics and Environmental Department, Transilvania University of Brasov, 35000 Brasov, Romania; 2 Renewable Energy Systems and Recycling Research Center, Transilvania University of Brasov, Eroilor 29 Street, 35000 Brasov, Romania; isac.luminita@unitbv.ro

**Keywords:** herbicide, s-metolachlor, wastewater, photocatalysis, semiconductors, S-scheme mechanism

## Abstract

A dual S-scheme Cu_2_S_TiO_2__WO_3_ heterostructure was constructed by sol–gel method using a two-step procedure. Due to the synthesis parameters and annealing treatment the heterostructure is characterized by sulfur deficit and oxygen excess allowing the passivation of oxygen vacancies. The photocatalytic activity was evaluated under UV and UV–Vis irradiation scenarios using S-MCh as reference pollutant. The heterostructure is composed on orthorhombic Cu_2_S, anatase TiO_2_ and monoclinic WO_3_ with crystallite sizes varying from 65.2 Å for Cu_2_S to 97.1 Å for WO_3_. The heterostructure exhibit a dense morphology with pellets and particle-like morphology closely combined in a relatively compact assembly. The surface elemental composition indicate that the heterostructure maintain a similar atomic ratio as established during the synthesis with a slight sulfur deficit due to the annealing treatments. The results indicate that the three-component heterostructure have higher photocatalytic efficiency (61%) comparing with two-component heterostructure or bare components. Moreover, Cu_2_S_TiO_2__WO_3_ exhibit a superior constant rate (0.114 s^−1^) due to the high concentration of photogenerated charge carriers, efficient charge separation and migration.

## 1. Introduction

Aquatic contamination with recalcitrant organic compounds released by industrial, agricultural, and transportation activities represent a serious threat to human health and life quality. Pesticide compounds including herbicides, insecticides, and fungicides have large contamination potential due to their continuous use in agricultural area and long term persistency in the ecological system [1,2,3]. The agricultural accumulation of organic compounds, such as S-metolachlor, cyprodinil, iprodione, tebuconazole, etc., in sediment and biota, are sources of serious environmental pollution [4,5]. Finding new and sustainable pathways for water decontamination represent an important target for the future of agriculture and wastewater plants. Photocatalytic technologies use the light energy as driving force to induce organic pollutant oxidation. The use of mono-component photocatalysts such as TiO_2_ [6], WO_3_ [7], SnO_2_ [8], Cu_2_S [9], CuO [10], or NiO [11] presents several disadvantages: (i) limited light absorption range, (ii) fast charge carriers recombination, and (iii) reduced chemical stability.

The photocatalytic process is based on three major steps: (1) the electron/hole pairs formation during irradiation due to the semiconductors absorption of photons with energy (hv) equal to or above their bandgap (Eg); (2) the separation and diffusion of photogenerated carriers under the action of the internal electric field; (3) formation of oxidative and super-oxidative species due to the surfaces reactions induced by the photogenerated electrons and holes [12,13,14]. TiO_2_ and WO_3_ have band gap values of 3.2 eV and 2.8 eV, which means that they are mostly active in the UV spectra. The charge carriers are photo-generated when the photons energy are equal or higher than the band gap value. Cu_2_S is photoactivated in Vis spectra due to his band gap value which may vary between 1.2 eV and 2.0 eV, depending on the stoichiometric composition. When coupled, the band gap values shift in order to generate a build in electric field required for charge carriers mobility through the heterostructure. Traditional heterojunctions (such as type-II heterojunctions, p-n junction) typically produce unfavorable losses of the photogenerated charges, while the Z-scheme heterostructure represent the starting point on the development of S-scheme heterostructure [15,16]. If compared with Z-scheme heterostructure, the S-scheme efficiently use the build-in electric field in order to reduce the migration distance of photogenerated electrons and holes based on the synergetic interface between the semiconductor components [17,18].

The S-scheme heterostructure consists of two or more semiconductors with suitable position of the energy bands. The driving force responsible of the charge migration is represented by the internal electric field established between the heterostructure components [19,20]. The photogenerated charges are separated in space, based on the semiconductors potential: the holes are located in the conduction band (CB) of the reduction photocatalyst and the electrons are in the valence band (VB) of the oxidation photocatalyst, respectively [21,22]. The photocatalytic reactions generating the (super)oxidative (•OH, •O_2_^−^) species are initiated by the photogenerated holes and electrons. Therefore, efficient S-scheme heterostructure is highly desirable, in order to promote the charge migration and to sustain favorable charge potentials [23,24]. Until now, several S-scheme heterostructures such as SnFe_2_O_4_/ZnFe_2_O_4_ [25], TiO_2_/W_18_O_49_ [26], NiO/BiOI [27], BiVO_4_@MoS_2_ [28], WO_3_/CdIn_2_S_4_ [29], CdS/UiO-66 [30] were tested for dyes or pharmaceutical active compounds removal. The SnFe_2_O_4_/ZnFe_2_O_4_ show enhanced Vis light absorbance and direct S-scheme path of charge separation and transfer. After 60 min of 300 W Vis light irradiation the NiO/BiOI exhibited 90% Rhodamine B photocatalytic removal.

Herein, we report for the first time the photocatalytic removal of S-MCh herbicide using a dual S-scheme heterostructure and two irradiation scenarios. The composition and morphology was investigated and correlated with the photocatalytic properties and energy band diagram. The dual S-scheme mechanism facilitates the photogenerated charge carriers migration and the efficient use of redox potential to induce pollutant degradation. Compared with Z-scheme mechanism, the S-scheme heterostructure use the build-in electric field to mitigate the charge transmission distance due to the synergic semiconductors interface. The S-scheme mechanism is able to use more efficiently the photogenerated charge carriers based on the charge density difference between the heterostructure components. The kinetic evaluation indicates a higher oxidation reaction rate for the three-component heterostructure compared with two-components or single component photocatalysts.

## 2. Materials and Methods

### 2.1. Photocatalyst Materials

#### 2.1.1. Preparation of Mono-Component Photocatalysts

Two powder components were prepared using sol–gel technique:

(i) Cu_2_S was prepared by mixing 0.2 mol of copper nitrate (Cu(NO_3_)_2_, 99.9%, Scharlau, Barcelona, Spain) aqueous solution with 0.5 mol of sodium thiosulfate (Na_2_S_2_O_3_, 99.9%, Scharlau) aqueous solution. After 15 min of stirring the gel was formed and kept 3 h undisturbed to achieve the complete precipitation. The precipitate was centrifuged and thermally treated at 120 °C in a ceramic capsule in sulfured (Sulfur, 99%, Sigma Aldrich, Munich, Germany) atmosphere.
(ii) WO_3_ was obtained by dissolving tungsten hexachloride (WCl_6_, 99.4%, Acros Organics, Geel, Belgium) in a mixture of ethanol (100%, Sigma Aldrich) and 2-propanol (100%, Sigma Aldrich). After 120 min of stirring a light yellow alcoholic solution was obtained. Then, 0.15 mol of natrium hydroxide (99.98%, Honeywell, Charlotte, NC, USA) was added drop by drop and the gel was formed. After precipitation and centrifugation the resulting powder was annealed for 8 h at 500 °C.

The TiO_2_ powder was purchased (99.99%, Scharlau, Barcelona, Spain) and used without any purification procedures.

#### 2.1.2. Preparation of Multi-Component Photocatalysts

Three bi-component and one three-component heterostructures were prepared by sol–gel technique:

(i) Cu_2_S_TiO_2_ sample was obtained using the same procedure as described for Cu_2_S, with the single modification that TiO_2_ powder was dispersed into copper nitrate solution, considering the Cu:Ti atomic ratio of 1:1. The final powder was thermally treated at 150 °C for 2 h.
(ii) Cu_2_S_WO_3_ sample was obtained following the procedure described for Cu_2_S. The WO_3_ powder already prepared was added into copper nitrate solution, considering the Cu:W atomic ratio of 1:1. The final powder was thermally treated at 150 °C for 1.5 h.
(iii) TiO_2__WO_3_ sample was prepared as using a similar procedure as presented for WO_3_, and the TiO_2_ power was dispersed into tungsten hexachloride solution. The uniform TiO_2_ distribution was assured by adding polyethylene glycol (99%, Scharlau) and the Ti:W atomic ratio was 1:1. The annealing treatment was done at 500 °C for 5 h.
(iv) Cu_2_S_TiO_2__WO_3_ sample was synthesized by adding TiO_2__WO_3_ powder, previously obtained, into copper nitrate solution and the mixture was stirred for 2 h. The Cu:Ti:W atomic ratio was 1:1:1. Then, 0.7 mol of sodium thiosulfate (Na_2_S_2_O_3_, 99.9%, Scharlau) was added drop by drop under continuous stirring. The precipitate was centrifuged and thermally treated at 150 °C for 2 h.

### 2.2. Photocatalytic Activity

The photocatalytic activity was tested using a closed reactor able to produce light irradiation from the top and lateral sides, which increase the radiation uniformity during the experiments. The reactor chamber use ventilation engines and thermocouples in order to preserve constant temperature (25 °C) and humidity (60%). Two kind of radiation sources were used (single or combined): 18W UVa black tubes (T8, 3Lx flux intensity, λ_UVa,max_ = 365 nm, range 310–390 nm, Philips) and 18W Vis cold tubes (TL-D Super 80/865, flux intensity 28Lx, λ_Vis,max_ = 565 nm, range 400–700 nm, Philips). Two irradiation scenarios where verified and the corresponding total irradiance is presented in Table 1.

S-Metholachlor (S-MCh) herbicide was used as reference pollutant molecule, due to his reluctance to traditional wastewater treatments procedures as well as his toxicity and accumulation potential into the biota. High concentrations of S-MCh may induce cytotoxicity and genotoxicity effect localized in human lymphocytes. The photocatalytic experiments were done using 30 mg/L S-MCh aqueous solution and the photocatalyst dosage was 30 mg/50 mL. Quart recipients were considered due to high UV transmittance. The total time of experiment was 10 h: 2 h in dark to reach the absorption equilibrium and 8 h under irradiation. The pollutant concentration was measured hourly and compared with the UV–Vis is calibration curve (274 nm is the S-MCh absorption wavelength). Finally, the photocatalytic efficiency was calculated considering the initial (*C*_0_) and final (*C*) concentrations based on the following Equation (1):

(1) $$\eta =\left[\frac{\left(C_{0}-C\right)}{C_{0}}\right]\cdot 100$$

### 2.3. Characterization Instruments

The presence of crystalline structure was evaluated using X-ray diffraction (XRD, Bruker, Model D8 Discover, Karlsruhe, Germany) with 0.003 degree scan step locked-couple technique and 0.015 s/step. The morphology characterizations were done with scanning electron microscopy (SEM, Hitachi model S–3400 N type 121 II, Tokyo, Japan) in high vacuum regime for mono-component samples and with field emission scanning electron microscopy (FESEM, SU8010, Fukuoka, Japan) operated at an accelerated voltage of 25 kV for multi-component samples. The light intensity and total irradiance values inside the photoreactor were measured using a class A high precision pyranometer (SR11, Hukselflux, Berlin, Germany). The optical properties as well as the photocatalytic activity were investigated using UV–Vis spectrometry (Perkin Elmer Lambda 950, Waltham, MA, USA).

## 3. Results and Discussions

### 3.1. Composition and Morphology

**Figure 1:**
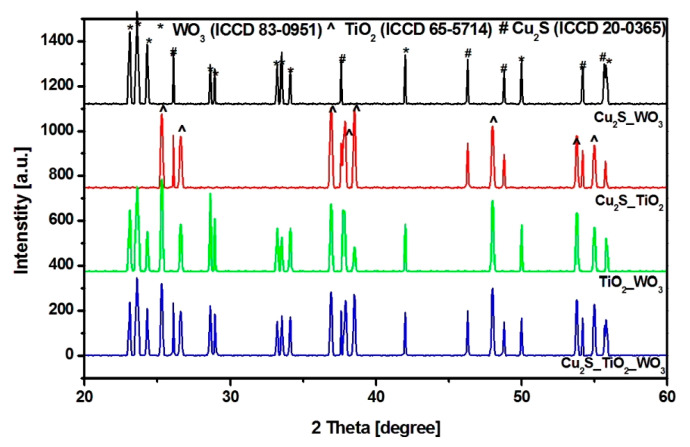
XRD patterns of the heterostructures.

The evaluation of crystalline structure presented in Figure 1 indicates that TiO_2_ has maintained the anatase structure (ICCD 83-0951) and crystallites size (Table 2), as received from the manufacturer, regardless the chemical and thermal treatments followed during the heterostructures development. The crystallite sizes were calculated based on the Scherrer formula, Equation (2):

(2) $$D=\frac{0.9\lambda }{\beta \cos\theta }$$

where *β* is the observed angular width at half maximum intensity (FWHM) of the peak, *λ* is the X-ray wavelength (1.5406 Å for CuKα1), and *θ* is the Bragg’s angle.

The anatase TiO_2_ structure is considered as more photosensitive compared with rutile and brookite, and suitable for photocatalytic applications. Cu_2_S exhibit orthorhombic structure (djurleite, ICCD 20-0365) and the crystallite size was influenced by the insertion of metal oxides during the sol–gel procedure. The presence of TiO_2_ favors the formation of smaller Cu_2_S crystallites size, while WO_3_ induce an increase of the copper sulfide crystallite sizes [31]. This behavior was previously presented by other researchers [32,33] in relation with similar materials. The insertion of metal oxides particles during the Cu_2_S development will act as preferential nucleation sites. Using larger metal oxide crystallites size will favor the grow process, which is no longer restricted by space limitation [34]. Contrary, if the nucleation sites are based on smaller metal oxides crystallites these will also limits the Cu_2_S crystallite grow rate [35]. The similar crystallites size values obtained for copper sulfide in Cu_2_S_TiO_2_ and Cu_2_S_TiO_2__WO_3_ samples is an indicator of the preferential Cu_2_S grow on TiO_2_ during the formation of three-component heterostructure. WO_3_ with monoclinic structure (ICCD 83-0951) show a similar behavior as Cu_2_S, and the crystallite size decrease in the presence of TiO_2_ during the sol–gel synthesis.

**Figure 2:**
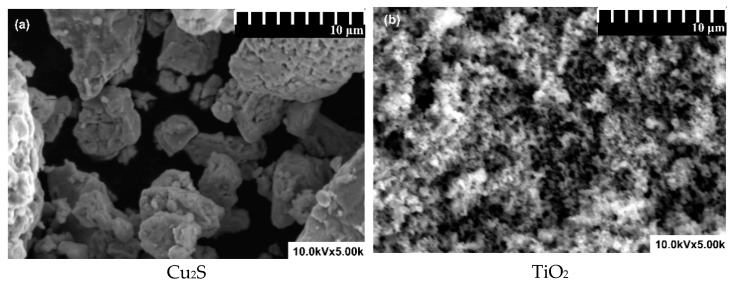

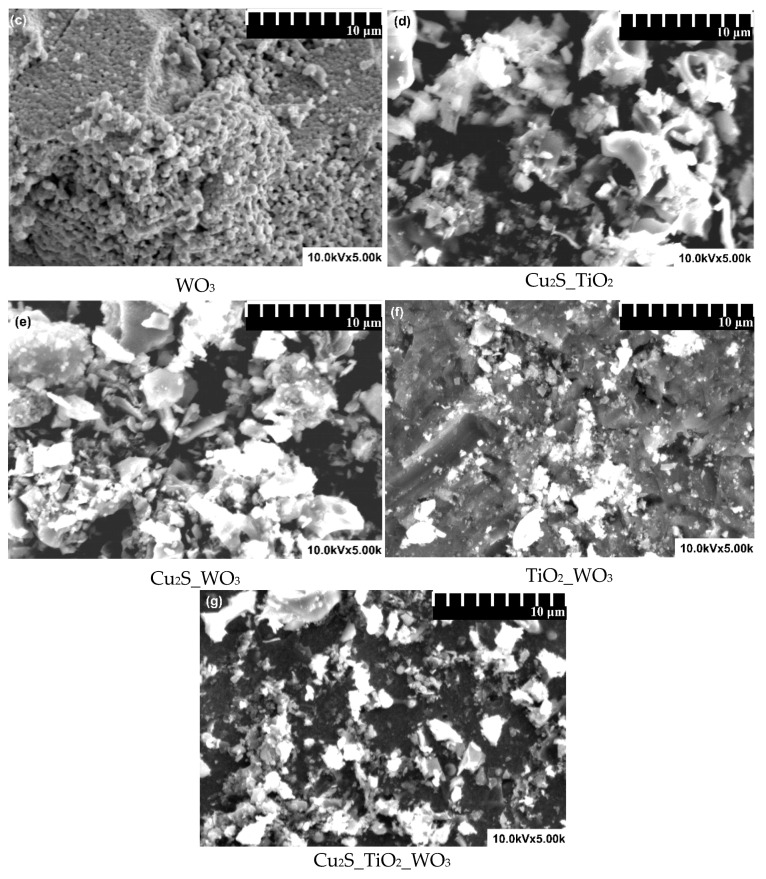
Scanning electron microscopy images of (**a**) Cu_2_S, (**b**) TiO_2_, (**c**) WO_3_, (**d**) Cu_2_S_TiO_2_, (**e**).Cu_2_S_WO_3_, (**f**) TiO_2__WO_3_, and (**g**) Cu_2_S_TiO_2__WO_3_.

The morphological analyses presented in Figure 2 indicate that both mono and multi-components samples are composed on particles with irregular shape and sizes. The mono-component samples contain smaller particles, which have the tendency to form larger aggregates. The average value of mono-component particles was 0.5 µm for Cu_2_S, 30 nm for TiO_2_ and 0.2 µm for WO_3_. The bi-component heterostructures containing Cu_2_S exhibit pellets and particle-like structures closely combined in a relatively compact assembly. The TiO_2__WO_3_ sample has a higher homogeneity due to use of polyethylene glycol (PG) additive which favor the uniform distribution of particles and decrease the aggregates size. A similar observation was made by Dudita M. [36], showing that the dispersive component of the PG can significantly decrease the aggregates formation. However, the PG removal procedure requires higher annealing temperature, in order to avoid carbon contamination, which is not suitable for samples containing Cu_2_S. Finally, the Cu_2_S_TiO_2__WO_3_ sample morphology indicates a uniform distribution of Cu_2_S on the TiO_2__WO_3_ assembly.

The EDS analysis was undertaken during the morphological investigations, in order to observe the elemental surface composition and the values are presented in Table 3. The results indicate that the atomic ratio between the metals was preserved as presented in the synthesis method. Small deviations of the atomic ratio for Cu_2_S_WO_3_ and Cu_2_S_TiO_2__WO_3_ may be attributed to the non-uniform distribution at the sample surface. Additionally, the qualitative results were compared with theoretical sulfur and oxygen content, calculated based on the stoichiometric compounds identified during the X-ray diffraction investigations. The values indicate a similar tendency of sulfur deficit and oxygen excess. The results are consistent with other studies [37,38], showing that the heterostructures submitted to annealing in air may lose sulfur and develop higher oxygen content. During the annealing treatment part of the oxygen vacancy formed during the synthesis procedure will be passivated as presented in Equation (3).

(3) $$V_{O}^{\prime \prime }+\frac{1}{2}O_{2}\to O_{O}^{x}+2h^{•}$$

### 3.2. Photocatalytic Properties and Mechanism

**Figure 3:**
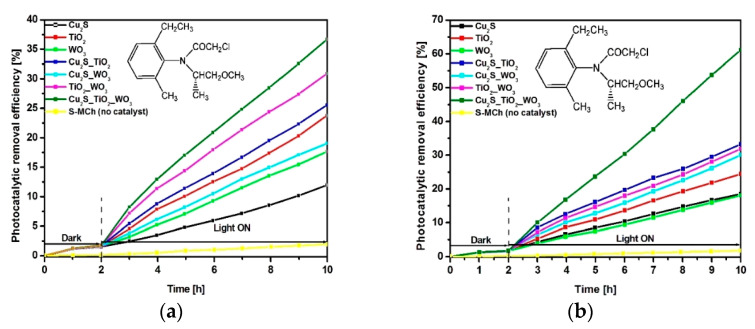
Photocatalytic removal efficiency under (**a**) UVa and (**b**) UVa–Vis irradiation.

The photocatalytic activity of the mono-component and multi-component samples was tested using two irradiation scenarios: UVa (Figure 3a) and combined UVa–Vis (Figure 3b). In all cases the S-MCh herbicide concentration was 30 mg/L and photocatalyst dosage was 30 mg/50 mL. As expected, under UV irradiation the samples containing TiO_2_ exhibit higher photocatalytic efficiency. The lowest photocatalytic efficiency (11.9%) was recorded for Cu_2_S sample which absorb mostly in Vis spectra. However, small photocatalytic efficiency differences where observed by comparing the Cu_2_S_TiO_2__WO_3_ (36.6%) and TiO_2__WO_3_ (30.8%) heterostructures, which means that the Cu_2_S contribution in UVa scenario is rather limited. This observation is valid for bi-component heterostructures containing Cu_2_S coupled with a TiO_2_ or WO_3_, where the photocatalytic efficiency increase with few percentage than that of mono-component samples. The combined UVa–Vis scenario brings the advantage of Cu_2_S component, which have a significant contribution in both bi and three-component heterostructures. The photocatalytic efficiency increase with additional 10% for Cu_2_S_TiO_2_ and Cu_2_S_WO_3_, comparing with UV scenario. The highest increase was observed for three-component heterostructure which reach 61.08% photocatalytic turnover rate, after 8 h of irradiation. These results are significant considering that S-MCh is considered to be a reluctant molecule towards photocatalytic decomposition due to the structural stability induced by the aromatic cycle and functional groups [39,40]. The formation of bi-products with pollutant potential cannot be excluded as partial oxidation may induce the development of carboxylic acids. Bare Cu_2_S, TiO_2_ and WO_3_ have lower photocatalytic efficiency due to the spectral absorption limitation and faster recombination charge. The experiments made without catalyst indicate a low S-MCh decomposition rate of 1.9% under UVa irradiation and 1.7% under UVa–Vis irradiations. The S-MCh ability of self-degradation in both irradiation scenarios is negligible.

**Figure 4:**
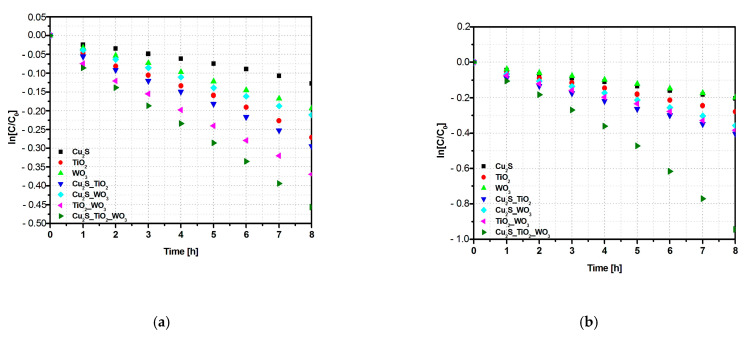
Kinetic evaluation of the photocatalytic activity under (**a**) UVa and (**b**) UVa–Vis irradiation.

The influence of photocatalysts composition and light irradiation scenario was further correlated with the kinetic investigation, based on the simplified Langmuir–Hinshelwood mathematical Equation (4) and the results are presented in Figure 4 and Table 4.

(4) $$\ln\frac{C}{C_{0}}=-kt$$

The kinetic data help to better understand the influence of the heterostructure composition on the photocatalytic activity. Under UVa irradiation the photocatalytic activity of Cu_2_S_TiO_2__WO_3_ heterostructure is 1.2x faster than TiO_2__WO_3_, 2.1x faster than Cu_2_S_WO_3_, and 1.5x faster than Cu_2_S_TiO_2_ bi-component samples. The differences increase significantly under UVa–Vis irradiation where the three-component sample photocatalytic activity is 2.5x faster than TiO_2__WO_3_, 2.7x faster than Cu_2_S_WO_3_, and 2.4x faster than Cu_2_S_TiO_2_ photocatalysts. It is worth noting that the differences between the constant rates of the bi-component heterostructures decrease in the combined irradiation scenario which confirms the contribution of Cu_2_S on the overall photocatalytic efficiency [41]. The kinetic evaluation made on bare Cu_2_S indicate that this component is 2x more active in UV–Vis scenario compared with UV, while TiO_2_ and WO_3_ exhibits small changes.

The L-H model (Equation (5)) proposed by Turchi and Ollis [42] was used to evaluate the influence of photon absorption based on both UV and UV–Vis irradiation scenarios.

(5) $$r=-\frac{dC}{dt}=\frac{k_{r}K_{S}C}{1+K_{S}C}$$

where *C* represents the S-MCh concentration (mol·L^−1^), *k_r_* correspond to the apparent reaction rate constant (mol·L^−1^·min^−1^), the apparent adsorption constant is represented by *K_s_* (L·mol^−1^) and the S-MCh removal rate is given by *r* (mol·L^−1^·min^−1^). It is useful to describe the apparent rate constant *k* (min^−1^) as *k_r_·K_s_* which is a specific term for each heterostructure photocatalytic activity. The *k_r_* depends on the light radiation parameters and allows changing the Equation (5), accordingly:

(6) $$\frac{1}{r}=\frac{1}{k_{r}K_{S}}\cdot \frac{1}{C}+\frac{1}{k_{r}}$$

The results obtained based on the linear plot 1/*C* vs. 1/*r*, considering the (1/*k_r_*) intercept and (/*k_r_K_S_*) slope are presented in Table 5. The mathematical model indicators show that L-H fits well with the experimental photocatalytic results obtained under UV–Vis irradiation. Similar results were obtained in UV scenario for TiO_2__WO_3_ and Cu_2_S_TiO_2__WO_3_ samples. These conditions allows that the apparent reaction rates have the same order of magnitude with the apparent adsorption constant, while for the Cu_2_S_TiO_2_ and Cu_2_S_WO_3_ heterostructures, there is one order of magnitude difference. There are similar reports in the literature [43,44], indicating the correspondence between the light irradiation spectra and the energy bands of the heterostructure composition which contributes to the photogeneration of electrons and holes and the conversion to oxidative species.

**Figure 5:**
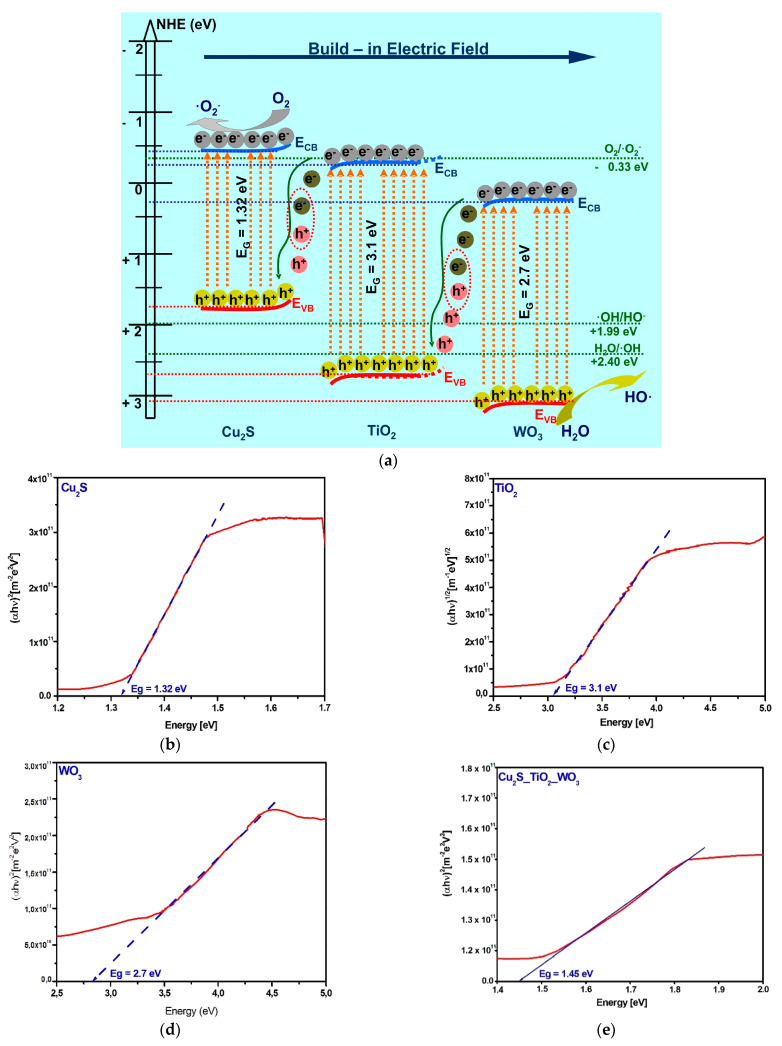
(**a**) Band energy diagram of the S-scheme heterostructure and the corresponding (**b**) Cu_2_S, (**c**) TiO_2_, (**d**) WO_3_, and (**e**) Cu_2_S_TiO_2__WO_3_ band gaps.

In order to evaluate the heterostructures mechanism, the components band energies (Figure 5) are necessary to provide additional information regarding the potential compatibility between them. The band energy diagram was developed based on the experimental band gap values (Figure 5b,c), corresponding to each heterostructure component and the bands energy potential were evaluated in good agreement with the methodology presented in literature [45,46]. The shift of band gap values in heterostructure may be possible due to the internal energy field. The procedure use the following Equations (7)–(10) to determine the energy band position based on the semiconductor electronegativity (*χ_semiconductor_*), energy of free electrons vs. hydrogen (*E_e_*), band gap energy (*E_g_*), absolute cationic electronegativity (*χ_cation_*), and specific cationic electronegativity $\chi_{cation}(P.u.)$ where *P.u.* represent the Pauling units.

(7) $$E_{VB}=\chi_{semiconductor}-E_{e}+0.5E_{g}$$

(8) $$E_{CB}=E_{VB}-E_{g}$$

(9) $$\chi_{semiconductor}(eV)=0.45\cdot \chi_{cation}(eV)+3.36$$

(10) $$\chi_{cation}(eV)=\frac{\chi_{cation}(P.u)+0.206}{0.336}$$

The bands energy diagram represents an S-scheme charge transfer mechanism corresponding to the heterostructures semiconductor components. Under light excitation the photogenerated electrons from the Cu_2_S conduction band (−0.4 eV) will migrate to TiO_2_ conduction band (Cu_2_S_TiO_2_ and Cu_2_S_TiO_2__WO_3_) and further on WO_3_ conduction band (+0.4 eV). However, the electrons from TiO_2_ and WO_3_ conduction bands (CB) cannot be involved in •O^−^_2_ generation, and the holes from Cu_2_S valence band (+0.92 eV) cannot produce •OH, owing to their potential. Consequently, some of those charges which are not useful for photocatalytic reaction will recombine [47,48]. The useful electrons from the Cu_2_S valence band (−0.4 eV), as well as the holes from TiO_2_ (+2.9 eV) and WO_3_ (+3.1 eV) valence bands, have a stronger redox ability and are efficiently separated by the electric field in the charged space region. Under the combined drift and diffusion effect, the photogenerated charges will migrate through the heterostructure components, [49,50]. The higher photocatalytic efficiency exhibited by Cu_2_S_TiO_2__WO_3_ is explained by the synergic activity of a dual S-scheme mechanism between the Cu_2_S/TiO_2_ and TiO_2_/WO_3_, respectively. The band gap values of each components shift, allowing the formation of a collective heterostructure band gap. The Tauc plot presented in Figure 5e indicate a band gap value of 1.45 eV for Cu_2_S_TiO_2__WO_3_ heterostructure, confirming the absorbance in both UV and Vis light spectra. Therefore, the development of S-scheme heterostructure systems could efficiently promote the photoexcited charge carriers separation which determine the increase of (super)oxidative species concentration acting on S-MCh photocatalytic removal.

## 4. Conclusions

A dual Cu_2_S_TiO_2__WO_3_ S-scheme heterostructure was prepared by sol-gel technique and the photocatalytic properties where tested and compared with other samples. The heterostructure is composed on orthorhombic Cu_2_S, anatase TiO_2_ and monoclinic WO_3_, with crystallite sizes varying from 65.2 Å for Cu_2_S to 97.1 Å for WO_3_. The heterostructure exhibit a dense morphology with pellets and particle-like morphology closely combined, in a relatively compact assembly. The surface elemental composition indicate that the heterostructure maintain a similar atomic ratio as established during the synthesis, with a slight sulfur deficit and oxygen excess due to the annealing treatments.

The photocatalytic activity toward S-MCh herbicide removal was evaluated using UVa and UVa–Vis irradiation scenarios. The results indicate a superior photocatalytic activity of Cu_2_S_TiO_2__WO_3_, compared with Cu_2_S_TiO_2_, Cu_2_S_WO_3_, TiO_2__WO_3_ or bare components. Under UVa–Vis irradiation the Cu_2_S_TiO_2__WO_3_ heterostructure reach 61% photocatalytic efficiency with a constant rate of 0.1140 s^−1^ (2.5x faster than TiO_2__WO_3_ and 2.7x faster than Cu_2_S_WO_3_). The higher photocatalytic activity of Cu_2_S_TiO_2__WO_3_ sample was attributed to the formation of dual S-scheme mechanism, allowing the increase of photogenerated charge carrier concentration, efficient charge separation and migration.

Future studies will be dedicated to the improvement of the photocatalytic efficiency using two approaches: (1) the optimization of heterostructure components ratio in order to increase the concentration of oxidative species, and (2) the replacement of one components in order to maximize the photons conversion during the photocatalytic activity. Both ways will preserve the S-mechanisms which seems to be the most favorable for resilient organic pollutants molecules. Additionally, HPLC studies will be included to investigate the formation of potential pollutant bi-products during the photocatalytic activity.

## Figures and Tables

**Table 1:** Irradiation scenarios for the photocatalytic applications.

Irradiation Sources	UV Radiation Sources (310–390 Nm)	Vis Radiation Sources (400–800 Nm)	Total Irradiance (W/M^2^)
UVa light, 18 W	8	0	16.3
UVa–Vis light, 18 W	4	4	24.9

**Table 2:** Average crystallite size values calculated using the Scherrer formula.

Samples Code	Crystallite Size (Å)		
	Cu_2_S	TiO_2_	WO_3_
Cu_2_S_TiO_2_	64	82	-
Cu_2_S_WO_3_	79	-	103
TiO_2__WO_3_	-	81	96
Cu_2_S_TiO_2__WO_3_	65	83	97

**Table 3:** Average atomic composition at the heterostructure surface (EDS).

Scheme	Elemental Composition (% at)						
	Cu	Ti	W	O	O_th_^1^	S	S_th_^1^
Cu_2_S_TiO_2_	23.7	22.6	-	45.4	45.2	8.3	11.8
Cu_2_S_WO_3_	14.2	-	16.7	62.5	50.1	6.6	7.1
TiO_2__WO_3_	-	13.8	12.1	74.1	63.9	-	-
Cu_2_S_TiO_2__WO_3_	14.3	9.8	9.6	59.9	48.4	6.4	7.1

^1^ Theoretic content calculated based on the stoichiometry.

**Table 4:** Kinetic data corresponding to S-MCh photocatalytic removal.

Kinetic Data	Cu_2_S		TiO_2_		WO_3_		Cu_2_S_TiO_2_		Cu_2_S_WO_3_		TiO_2__WO_3_		Cu_2_S_TiO_2__WO_3_	
	K [S^−1^]	R^2^	K [S^−1^]	R^2^	K [S^−1^]	R^2^	K [S^−1^]	R^2^	K [S^−1^]	R^2^	K [S^−1^]	R^2^	K [S^−1^]	R^2^
UV	0.0148	0.9958	0.0316	0.9964	0.0235	0.9992	0.0346	0.9971	0.0256	0.9984	0.0435	0.9966	0.0540	0.9977
UV–Vis	0.0243	0.9962	0.0334	0.9961	0.0237	0.9972	0.0469	0.9949	0.0419	0.9967	0.0448	0.9964	0.1140	0.9899

**Table 5:** Heterostructures kinetic parameters for both radiation scenarios based on Equation (6).

Photocatalyst, Pollutant	k_r_·10^8^ (Mol·L^−1^·Min^−1^)	K_s_·10^2^ (Mol·L^−1^)
Cu_2_S_TiO_2_, UVa	1.63	624
Cu_2_S_TiO_2_, UVa–Vis	2.94	1682
Cu_2_S_WO_3_, UVa	1.42	556
Cu_2_S_WO_3_, UVa–Vis	2.98	1625
TiO_2__WO_3_, UVa	2.68	1044
TiO_2__WO_3_,UVa–Vis	2.73	1592
Cu_2_S_TiO_2__WO_3_, UVa	3.19	1788
Cu_2_S_TiO_2__WO_3_, UVa–Vis	5.26	2389

## Data Availability

Data presented in this study are available by requesting from the corresponding author.
